# Exposure to Sleep, Rest, or Exercise Impacts Skill Memory Consolidation but so Too Can a Challenging Practice Schedule

**DOI:** 10.1523/ENEURO.0198-21.2021

**Published:** 2021-09-09

**Authors:** Taewon Kim, David L. Wright

**Affiliations:** 1 Department of Neurology, Duke University School of Medicine, Durham, NC 27710; 2 Department of Kinesiology, Texas A&M University, College Station, TX 77845

**Keywords:** consolidation, contextual interference, interleaved practice, motor learning, procedural memory

## Abstract

When discussing procedural learning, it is now routine to consider both online and offline influences for skill acquisition. This is because it is commonly assumed that the evolution of a novel skill memory continues well after practice is over. Indeed, factors impacting offline contributions to skill memory development such as sleep and exercise have garnered considerable research interest in recent years. This is partly because of their capacity to foster postpractice consolidation, a process that has been identified as critical to moving a skill memory from a labile to more stable or elaborate form. While uncovering the potency of non-practice factors to facilitate consolidation is undoubtedly important, the present opinion is designed to remind the reader that a practice schedule, organized to challenge the learner, can, in and of itself, be effective in supporting consolidation resulting in significant gains in long-term skill retention.

## Significance Statement

Adopting “best practices” is an objective for training and rehabilitation design and typically focuses on practice composition. Recent work has championed postpractice activities, such as sleep and exercise, as critical to the evolution of skill memory. Such practice adjuncts presumably facilitate skill acquisition by improving memory consolidation. The present opinion highlights the potency of practice organization as a means to also improve postpractice consolidation illustrated by findings revealing that experience with an interleaved rather than repetitive practice (IP) format results in performance gains for up to 72 h following the conclusion of training. Such latent gains are associated with heightened primary motor cortex (M1) excitability following practice in a manner similar to that observed when supplementing practice with sleep or exercise.

## Fostering Skill Memory through Rest, Sleep, and Exercise

Most accept that the evolution of a skill memory continues long after practice is over (Robertson and Takacs, 2017). Early efforts to look beyond the boundaries of practice to finds ways to enhance retention of a new skill focused almost exclusively on unique contributions from sleep and wake periods (Robertson et al., 2004). Indeed, a recent report of micro-consolidation during skill acquisition has reignited interest in the rest period during wake as the temporal locus of critical memory processes central to learning even when the interval is just a few seconds (Bonstrup et al., 2019). More recently, a quite different intervention, a brief bout of cardiovascular exercise, has also been touted as a powerful supplement to practice capable of procuring latent gains in skill (Jo et al., 2019; Chen et al., 2020).

In general, facilitation in skill memory following a sufficient rest period, exposure to sleep, and/or exercise is proposed to result from improved memory consolidation. The behavioral manifestation of successful consolidation is preservation of performance, even in the face of interference, or a gain in performance across a significant time period despite no additional practice. A vast body of evidence exists that reveals a distinction between consolidation that is sleep or time dependent leading to significant gains for a wide-range of procedural skill memories. In the case of sleep, novel skill memories can be facilitated when privy to either overnight sleep or a daytime nap (for recent review, see King et al., 2017). In contrast, the case for using acute exercise as a mediator of postpractice consolidation is still in relative infancy. Despite this, evidence is currently available supporting the claim that a brief bout of vigorous cardiovascular activity can protect a newly acquired procedural memory from interference from subsequent procedural (Jo et al., 2019) or declarative (Chen et al., 2020) learning that occurs in close temporal proximity. Moreover, when the interfering activity is absent, the administration of an acute bout of exercise immediately after novel skill learning, rather than an equivalent period of rest, has been reported to enhance skill memory assessed across an 8-h wake period (Ostadan et al., 2016).

## Increasing Interference during Practice Can Facilitate Consolidation of Novel Skill Memories

While the use of sleep and exercise as adjuncts to practice continue to capture the research community’s attention, the importance of practice itself, arguably the most critical determinant of the development of skill memory, has taken a relative backseat. The present commentary is an attempt to remind the reader that organizing practice of a novel skill can also be a conduit to continued processing of a newly formed skill memory well beyond the conclusion of a bout of physical practice. In the present case, the potency of one particular practice organization for encouraging postpractice consolidation is highlighted by briefly reviewing some recent data addressing contextual interference (CI), a practice scheduling phenomenon that has a rich history in the motor skill literature (Wright et al., 2016). Thus, rather than manipulate the postpractice period directly (i.e., by experiencing sleep or exercise after practice), here we demonstrate that the arrangement of practice can be such that postpractice memory processing is encouraged that can lead to sizeable latent gains.

**Figure 1.**
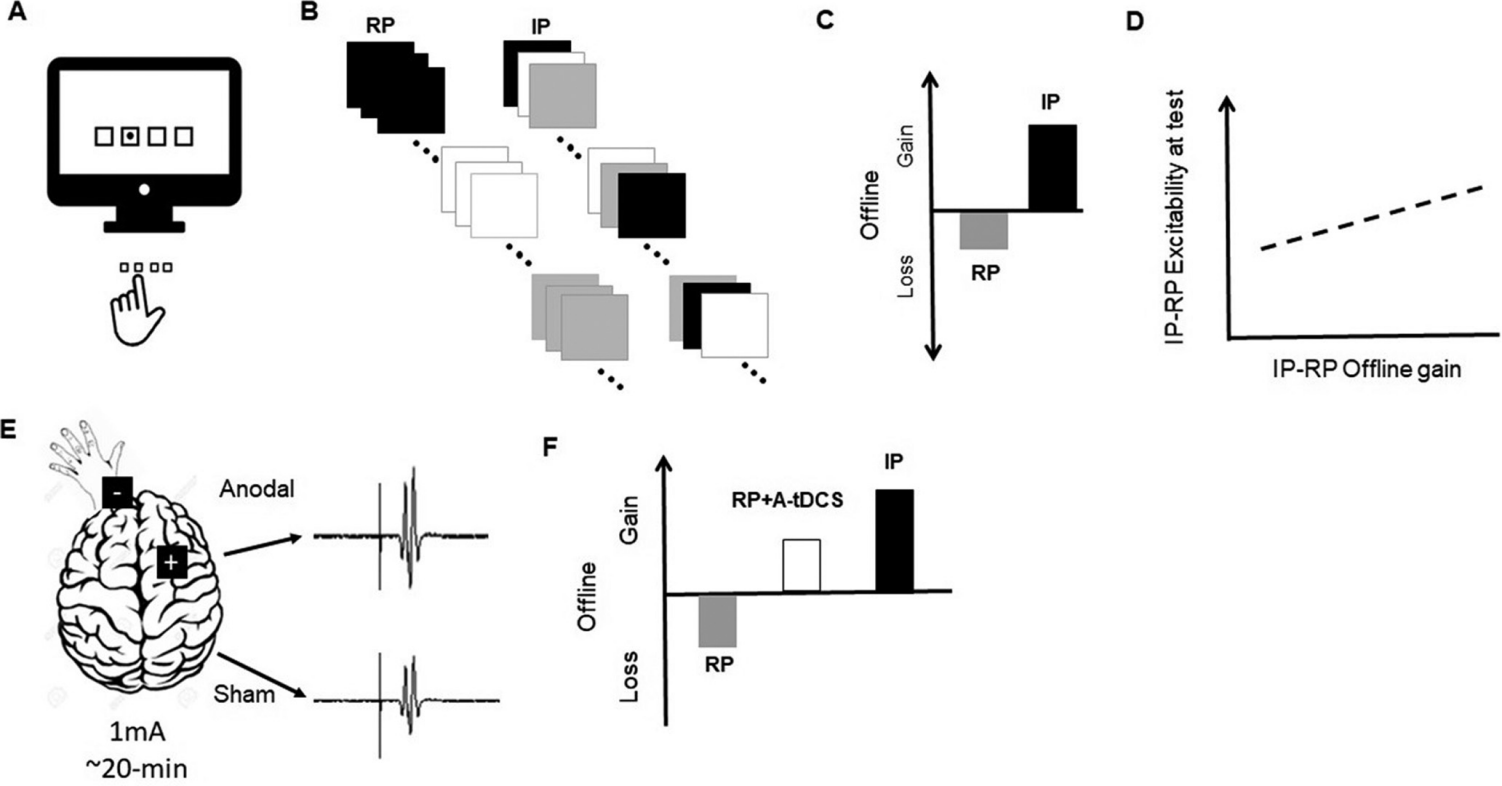
A motor sequence task is a common procedural skill used to reveal offline performance gains. This task requires the performer execute a series of key presses that are spatially compatible with visual signals presented on a display as quickly and accurately as possible (***A***). A number of motor sequences (trials for three unique sequences, represented in black, gray, or white, are used for the purpose of illustration in this example) are practiced in either a repetitive (RP) or interleaved (IP) format during a period of training. For RP, all trials of practice for one of the sequences are completed during a period of training before any practice with an alternative motor sequence is introduced. Alternatively, in IP, during any period of practice, an equal number of trials for all of the to-be-learned sequences are experienced. However, the presentation of trials of practice for these to-be-learned sequences rarely, if ever, involve the execution of the same sequence on two consecutive trials within a single bout of training. The impact on performance following each of these training contexts is most commonly assessed during a delayed test usually administered 24–72 h later (not shown in diagram; Lin et al., 2011; Wright et al., 2016; ***B***). Offline gain, defined as the difference between performance at the time of test and at the end of training in either a IP or RP format, is greater following IP compared with RP (Wright et al., 2016; Lin et al., 2011; ***C***), and the difference in offline gain achieved following RP and IP is related to the difference in cortical excitability observed at M1 before a test administered 72 h after practice was completed between individuals assigned to IP and RP (Lin et al., 2011; ***D***). tDCS involves the passage of direct current between two electrodes [anode (+), cathode (–)]. Anodal stimulation (e.g., 1 mA for 20 min) is a condition in which the anode is placed over the neural region being targeted resulting in an upregulation of activity at the stimulated site (e.g., M1) reflected in an increased MEP from the application of single-pulse TMS compared with sham stimulation (***E***). Applying anodal tDCS at right M1 while the learner performed a motor sequence task with the left hand during RP results in offline gains rather than the anticipated loss in performance commonly observed following RP when performed in the absence of stimulation (Kim and Wright, 2020; ***F***).

Experimental evidence from studies conducted under the rubric of CI underscore the importance of how practice organization can influence the resultant long-term retention of newly acquired skill memories. The CI phenomenon has most commonly focused on learning outcomes that result from the simultaneous acquisition of multiple novel procedural skills in either an interleaved practice (IP) or repetitive practice (RP) format (Fig. 1*A*,*B*; Wright et al., 2016). IP is argued to induce greater demand on motor planning operations during training because of the frequent change in the trial-to-trial task demands that are encountered by the learner during this practice format (Li and Wright, 2000). Specifically, when an individual learns in the context of IP, they rarely, if ever, execute the same procedural skill on two consecutive practice trials within a single bout of training. Conversely, RP, a commonly adopted protocol in many instructional and rehabilitation settings, is argued to create relatively less interference because it involves the repeated execution of the same motor task before encountering any practice with other motor tasks that will also be practiced, and hopefully learned, during a single practice episode.

It turns out that IP, while characterized by poorer encoding reflected in a relatively slower rate of skill acquisition but greater attention demand assessed using dual-task procedures (Li and Wright, 2000), is more effective for supporting long-term retention of novel skill memory most often assessed 24 h after practice is over (Shea and Morgan, 1979; for limitations of IP, also see Wulf and Shea, 2002; Russell and Newell, 2007). Of particular interest for the present discussion is the observation that individuals exposed to IP, much like the participants privy to exercise or sleep following practice, exhibit a dramatic improvement in performance across a 24- to 72-h test interval despite the absence of any extra physical practice, that is, they display offline enhancement (Lin et al., 2011; Wright et al., 2016; Fig. 1*C*). What makes this gain even more impressive is that it emerges even when the test context for IP-trained learners is incongruent with the practice format that was experienced during original learning. That is, the gains after practice are equally likely to surface if the test of skill memory is administered in a RP or IP format. For example, recent work revealed more than a 20% loss in performance 48 h after the conclusion of RP compared with a >30% gain in performance over the same interval following IP when test trials were administered in a RP schedule (Kim et al., 2018). Thus, skill memories resulting from exposure to IP are not only enhanced but are quite robust to a change in performance context. On the other hand, participants that experience RP, despite exposure to the same total number of practice trials within a single training episode exhibit significantly better performance when the test environment matches that encountered during acquisition (Lin et al., 2011).

The robustness of skill learning following IP as opposed to RP is further highlighted by findings that demonstrate that the offline gains fostered by IP exhibit some resistant to interference from supplemental practice with another novel procedural skill. Specifically, despite the additional practice with a new procedural skill that began 5 min after IP was completed, participants still managed to exhibit performance 24 h later that was similar to that achieved at the end of training. This was not the case for their RP counterparts who revealed substantial degradation in performance across the same timeframe (Kim et al., 2016). These data are reminiscent of the protective effect provided by exposure to sleep or exercise following the formation of a newly developed skill memory. For example, participation in a brief bout of cardiovascular exercise immediately after practice of a novel motor skill safeguards this new memory from a subsequent bout of training with another untrained motor skill that in the absence of the exercise will induce significant retroactive interference sufficient to eliminate memory for the initial skill (Jo et al., 2019).

Finally, it is critical to highlight that offline enhancement garnered via IP is not restricted to retention that is evaluated after 24 h or longer during which the learner is privy to overnight sleep as is the case for the influence of acute exercise for offline gain (Jo et al., 2019). Performance facilitation from IP has also been observed during tests administered as early as 6 h following the termination of practice as well as during the more common time frames used to assess memory, typically up to 72 h, when sleep-related consolidation might also contribute to any reported benefits (Kim and Wright, 2020). These data are important because they implicate practice organization, specifically IP, as a means to mediate postpractice consolidation independent of the powerful impact induced from exposure to sleep.

Taken as a whole, it is difficult to ignore the observation that interleaving practice of multiple novel skills as well as experiencing a period of sleep (King et al., 2017) or exercise (Robertson and Takacs, 2017) are effective means of fostering memory consolidation that can improve long-term skill retention. On the basis that functional benefits can emerge for each of these interventions, it may not be that surprising that evidence is emerging that suggests there may be some mechanistic overlap through which consolidation is implemented following IP, sleep, and/or exercise. These data are explored in the next sections.

## Consolidation of Skill Memory Involves Neural Circuitry That Includes the Primary Motor Cortex (M1)

Consolidation responsible for the emergence of offline gain in the performance of a novel motor skill, has been argued to be dependent on, or at least involve, heightened activation of neural circuitry that includes the M1 (Robertson and Takacs, 2017; also see Mirdamadi and Block, 2020). This is certainly true in the case of early consolidation that is argued to occur in the first few hours after practice is complete. A recent proposal claims that M1 excitability shortly after the completion of procedural skill practice can be used as a physiological marker signifying that the appropriate neural machinery is in place to support offline improvement (Tunovic et al., 2014). This work demonstrated that when corticospinal excitability, typically assessed using the size of the motor-evoked potential (MEP) of the end-effector (e.g., first dorsal interosseous; FDI) induced by single-pulse transcranial magnetic stimulation (TMS) at M1, is temporarily downregulated immediately after practice, no offline gain is evident across a wake period. However, application of a theta-burst TMS protocol at this same neural site immediately after practice, designed to exogenously elevate corticospinal excitability, was sufficient to support offline improvement.

The aforementioned data have been used as the basis for a causal link between the state of M1 at the conclusion of training and the likelihood that offline gains will emerge from an early form of consolidation (Tunovic et al., 2014). This claim is further supported by evidence of delayed performance enhancement via the direct upregulation of M1 using non-invasive stimulation during practice of a novel skill (Reis et al., 2009). Critically, for the present discussion, the effectiveness of sleep (Xu et al., 2019) and exercise (Ostadan et al., 2016) for promoting consolidation and associated increased offline gain has also recently been associated with upregulation of cortical excitability at M1.

In keeping with the proposal that an elevation in excitability at M1 is related to the implementation of consolidation and the concomitant offline gain, there is also evidence revealing that relative greater activity of M1 as a significant contributor to the skill learning advantage garnered from IP over RP. Specifically, activity at M1 during response preparation is greater and occurs across a more prolonged period during a period of IP compared with RP (Lin et al., 2011). TMS-induced MEPs at the time skill memory is assessed (i.e., during delayed retention tests) are of relatively greater magnitude for learners trained with IP as opposed to RP and importantly, the magnitude of the difference in cortical excitability as a function of practice schedule correlates with extent of the offline improvement benefit observed from IP (Shea and Morgan, 1979; Lin et al., 2011 see also Tunovic et al., 2014; Fig. 1*D*). Moreover, applying supra-threshold single-pulse TMS at M1 following practice trials during IP, used to disrupt processes occurring at M1 during training, attenuates the offline gain associated with this practice format (Lin et al., 2010). Finally, upregulating the activity at M1 during RP, via administration of anodal transcranial direct current stimulation (tDCS), can induce offline gains that are absent for participants that trained in an RP training condition but was paired with sham tDCS (Fig. 1*E*,*F*; Kim et al., 2021).

It is important to note these data do not necessarily imply that the neural substrates supporting the effects of these different interventions are (1) restricted to just M1 or (2) involve the same neural substrates. Indeed, there are recent data indicating that exogenous stimulation using tDCS of neural sites beyond M1 during RP can mediate the nature of offline changes in performance (Kim and Wright, 2020). At this juncture then, the extant data suggest that, much like the case for sleep and exercise, the implementation of consolidation following IP that moves skill memory from its initial labile to more stable or even enhanced form is linked to heightened activity at M1 or maybe more broadly in neural circuitry incorporating M1. Deciphering whether this occurs from local effects at M1 or from remote influences impinging on M1 and whether these neural dynamics coincide across sleep, exercise, and practice organization will require additional experimental attention in the future.

## Final Thoughts

Identifying training methods to foster successful long-term retention while also inducing some capability for generalization is central to research focused on the development of skill memory. Significant effort of late has been exerted in demonstrating the efficacy of “non-practice” features of the learning environment that can contribute to the continued evolution of skill memory following initial encoding. This is best reflected in the current-day interest in the role of sleep, and to a lesser extent exercise, as a means of fostering postpractice consolidation sufficient to improve offline gains in skill memory.

Addressing the potency of non-practice factors for fostering consolidation is undoubtedly important highlighting the need to expand our understanding of the entire context to which newly acquired skill memories are exposed including those that exist beyond the traditional boundaries of practice (King et al., 2017; Robertson and Takacs, 2017). This may be an especially important message on a practical level as traditionally exclusive emphasis is placed on the training or rehabilitation session itself when contemplating “best-practice.” Moreover, new findings from studies addressing the contribution of sleep and exercise to offline gains in skill continue to help refine the initial mechanistic accounts of procedural skill consolidation at this time focused on the role of circuits involving M1 (King et al., 2017; Robertson and Takacs, 2017; Mirdamadi and Block, 2020). Despite these advances, the present discussion reminds us that increasing our awareness of how practice is organized, may not be such a bad thing, as it too, can foster consolidation of skill memory not afforded by other practice schedules (Wright et al., 2016; also see Hesseg et al., 2016).

## Synthesis

Reviewing Editor: Morgan Barense, University of Toronto Decisions are customarily a result of the Reviewing Editor and the peer reviewers coming together and discussing their recommendations until a consensus is reached. When revisions are invited, a fact-based synthesis statement explaining their decision and outlining what is needed to prepare a revision will be listed below. The following reviewer(s) agreed to reveal their identity: NONE.

SYNTHESIS:

This opinion piece provides parallels between memory consolidation processes which have mainly been studied through post-practice methods and those related to the actual practice conditions. Although the type of practice identified is not a novel method (i.e., CI), the systematic application of this method to study offline learning advances understanding of when and how this takes place.

Although the reviewers were enthusiastic about the overall contribution of the work, each highlighted specific concerns and suggestions (provided below). We hope these are helpful as you revise your manuscript.

REVIEWER 1:

The goal of this opinion paper is to remind the scientific community about the importance of the effect of practice schedule on motor performance. The authors summarise a series of contextual interference (CI) studies showing the advantage of interleaved as compared to repeated practice (IP vs. RP). Results are compared to findings from research investigating the effect of sleep and exercise on motor performance. While I fully agree with the authors that the CI topic is definitely of interest in the motor learning / motor memory consolidation field, I have some concerns listed below.

First, it is unclear why the authors focused on a comparison between the effect of sleep/exercise and the effect of CI on offline gains in performance as there are several other interventions like e.g. non-invasive brain stimulation applied during learning or during consolidation that have been extensively studied in the context of motor memory consolidation. Additionally, an important concept is missing in the opinion as sleep/exercise interventions for example have mainly been used as a way to enhance the offline consolidation process while CI is based on a manipulation of initial encoding. Putting these particular interventions in opposition in the paper seems a bit arbitrary. Altogether, I am not convinced about the angle of the opinion (CI is as good as sleep/exercise) and I am not sure such angle is necessary to highlight the value of CI.

Related to this, I would recommend to avoid making quantitative statements such as “appropriately scheduled practice can lead to gains in motor performance that are similar to, or in some cases exceed, those previously described from exposure to sleep and/or exercise” or compare the magnitude of offline gains across interventions (e.g., sleep vs. IP) as such statements are not supported by experimental data or based on the results of meta-analysis comparing the outcome of the different interventions. Additionally - and this point should also be addressed in the opinion - IP might induce larger offline performance gains as compared to other interventions because of baseline differences in performance induced by interference (initial performance being lower, there is more room for offline gains in performance to emerge). Importantly, performance at retest might reach similar levels between interventions. Therefore, I would recommend to move away from these direct comparisons but rather keep the discussion focused on comparisons between RP and IP.

In the same vein, it is rather expected that intervention modulating motor learning and motor memory consolidation would involve neural circuits including M1. It does not necessarily imply that the neural substrates supporting the effect of these different interventions are similar.

Last, none of the limitations of CI experiments are mentioned in the opinion. This information will be of great interest for the reader. I would recommend adding a limitation section that highlights eg, how some of the previous CI research might have been confounded by eg. practice order effect (Sherwood & Lee, 2003).

Minor points:

There are multiple typos in the text. A few examples are listed below:

- Line 12: resulting is significant gains

- Line 16: post-practice consolidation manifest as offline performance gain

- Line 211: attention to the nature of practice itself, in particular the manner in which practice is organized, may not be such a bad thing, as it to, can foster ...

Some sentences are rather unclear and would benefit from revisions:

- Line 21: Offline gain from improved consolidation from offline influences such as sleep, exercise, as well as online manipulations such as interleaved rather than repetitive practice is related to increased cortical excitability in neural circuits that include M1.

- Line 144: Mechanistically, consolidation that is assumed to be responsible for offline gain in novel motor skill, has been argued to be dependent on, or at least involve, neural circuitry that includes, but probably not limited to [e.g., see (Mirdamadi & Block, 2020)], the primary motor cortex (M1) (Robertson & Takacs, 2017)

REVIEWER 2:

This is a well-written, interesting opinion piece on potential similarities between conditions which promote offline learning... I just had a few question/observations.

• I think it would help to have clearer distinctions/clarity between practice interventions and post-practice interventions. It appears from the outset that the opinion paper is about post-practice interventions and that these are designed to augment the “learning” that took place during the practice episode and presumably is still continuing. An intervention such as sleep, rest, exercise or stimulation is therefore a supplement to the quality and/or quantity of the initial practice episode. When the idea of effective practice organization is introduced after discussion of post-practice interventions, this distinction then gets blurred (l63>).

• Related to this point of clarity above, is offline enhancement from “good quality” practice observed with other conditions of practice beyond CI? What is the critical ingredient that likely leads to offline gains? I appreciate the later discussion of M1 activation as mechanisms, but can the authors speculate on the actual method or quality of practice itself that is needed (perhaps beyond the CI effect)?

• It might help the reader to give some description as to how offline gains and losses are calculated in a CI paradigm in view of the different types of practice and the fact that the end of the practice session reflect different sequences for IP and RP groups. These gains are still interesting, but is it because performance has not plateaued for the IP groups in comparison to the RP groups? Are offline gains still seen if the same level of accuracy is attained at the end of practice by these different groups? Are gains ever inferred from differences in immediate and delayed retention tests where the different groups are tested under the same conditions?

• In discussing the mechanistic similarities between post-practice unrelated task methods and practice methods themselves, is it simply the case that any method which excites M1 “sufficiently” in practice or post-practice will produce offline learning? Does offline learning occur for cognitive tasks that do not involve action components and presumably no M1?

MINOR

l12; “in"

l29 “a new skill” or “new skills"

l59-63... this is a long and difficult sentence

l294: include “hours” in the figure heading after 24-72.

Figure 1: It would be useful to explain what “test” means in the relationship figure between offline gain and cortical excitability. Is this excitability after 24 or 72 hours of not practising, just as an individual is getting ready to execute the sequences in a retention phase, or during the retention phase?

L114: change “than” to “that"

L133: “taken as a whole...”

L133>136: this is also quite a long difficult sentence that would benefit from parsing/rephrasing.

L161: given that this is a new paragraph here, it would help to specify what “these data” refer to in the first line.

L164: when you say “learning” here, can you be more specific, do you mean practice or post practice?

L190-193: can this sentence be parsed/simplified too?

L212: maybe structures is not the best word here as it can imply brain areas... perhaps methods or schedules?

## Author Response

Synthesis of Reviews:

Significance Statement Comments for Author (Required):

With some minor revisions, the Significance Statement could be made more accessible and exciting for researchers not in this field. I encourage the authors do this.

Authors: We agree. The significance statement has been completely re-written with the goal of highlighting the key points in a manner more meaningful to interested individuals outside the field of motor neuroscience.

Comments on the Visual Abstract for Author (Required):

N/A

Synthesis Statement for Author (Required):

SYNTHESIS:

This opinion piece provides parallels between memory consolidation processes which have mainly been studied through post-practice methods and those related to the actual practice conditions. Although the type of practice identified is not a novel method (i.e., CI), the systematic application of this method to study offline learning advances understanding of when and how this takes place.

Although the reviewers were enthusiastic about the overall contribution of the work, each highlighted specific concerns and suggestions (provided below). We hope these are helpful as you revise your manuscript.

REVIEWER 1:

The goal of this opinion paper is to remind the scientific community about the importance of the effect of practice schedule on motor performance. The authors summaries a series of contextual interference (CI) studies showing the advantage of interleaved as compared to repeated practice (IP vs. RP). Results are compared to findings from research investigating the effect of sleep and exercise on motor performance. While I fully agree with the authors that the CI topic is definitely of interest in the motor learning / motor memory consolidation field, I have some concerns listed below.

Authors: Thanks for the positive comments regarding the manuscript and taking the time to identify issues that need to be addressed to improve this work.

First, it is unclear why the authors focused on a comparison between the effect of sleep/exercise and the effect of CI on offline gains in performance as there are several other interventions like e.g. non-invasive brain stimulation applied during learning or during consolidation that have been extensively studied in the context of motor memory consolidation. Additionally, an important concept is missing in the opinion as sleep/exercise interventions for example have mainly been used as a way to enhance the offline consolidation process while CI is based on a manipulation of initial encoding. Putting these particular interventions in opposition in the paper seems a bit arbitrary. Altogether, I am not convinced about the angle of the opinion (CI is as good as sleep/exercise) and I am not sure such angle is necessary to highlight the value of CI.

Authors: The primary goal is of course to reiterate and/or highlight the fact that the actual practice format can have an impact on memory development after practice is complete by impacting processes that occur after practice is complete. The motor learning community has addressed the influence of IP quite extensively at the behavioral level recognizing differential post-practice dynamics when compared to RP. The broader neuroscience community’s interest in the role of consolidation for skill acquisition has focused primarily on direct manipulations during the post-practice period with sleep being probably one of the most examined features in recent history. Reviewer 1 is of course correct that attempts to more directly impact post-practice activity also includes work using non-invasive stimulation (e.g., tDCS). We considered including this approach (in addition to sleep and exercise) but decided against this partly because applying tDCS whilst practicing as opposed to during the post-practice period has been reported to be more effective (we have published some recent work addressing this issue and there are also a number of papers from Janine Reis’ group demonstrating this outcome). We wished to highlight practice structure (an online manipulation or encoding if you prefer) that could instigate subsequent consolidation rather than the more studied methods that have been used to target this process more directly (i.e., offline). Indeed, what Reviewer 1 argued is missing we feel is the essence of the paper and was a point that Reviewer 2 wanted to be note explicitly in the paper.

As a result we now state “In the present case, the potency of one particular practice organization for encouraging post-practice consolidation is highlighted by briefly reviewing some recent data addressing contextual interference (CI), a practice scheduling phenomenon that has a rich history in the motor skill literature [8]. Thus, rather than manipulate the post-practice period directly (i.e., by experiencing sleep or exercise after practice), here we demonstrate that that the arrangement of practice can be such that post-practice memory processing is encouraged that can lead to sizeable latent gains.” (At the end of the section entitled - Increasing interference during practice can facilitate consolidation of novel skill memories)

Related to this, I would recommend to avoid making quantitative statements such as “appropriately scheduled practice can lead to gains in motor performance that are similar to, or in some cases exceed, those previously described from exposure to sleep and/or exercise” or compare the magnitude of offline gains across interventions (e.g., sleep vs. IP) as such statements are not supported by experimental data or based on the results of meta-analysis comparing the outcome of the different interventions.

Authors: We completely agree with Reviewer 1 regarding the use of the noted statements. In fact, our goal is not to argue/claim that particular practice scheduling offers “greater” benefits for consolidation than any other manipulations. Rather we wish to highlight the potential of manipulating practice per se as a way to induce consolidation which has been shown to be so critical to learning and memory - a finding most commonly based on studies directly modify the implementation of consolidation - we selected two popular approaches - sleep or exercise - to make this point.

We have hopefully removed the most egregious examples of comparing the magnitude of offline gain from IP to that obtained from sleep and or exercise (i.e., at the beginning of the section entitled - Increasing interference during practice can facilitate consolidation of novel skill memories.

Additionally - and this point should also be addressed in the opinion - IP might induce larger offline performance gains as compared to other interventions because of baseline differences in performance induced by interference (initial performance being lower, there is more room for offline gains in performance to emerge). Importantly, performance at retest might reach similar levels between interventions. Therefore, I would recommend to move away from these direct comparisons but rather keep the discussion focused on comparisons between RP and IP.

Authors: Point taken and we have tried to review the manuscript to remove any such direct comparison.

In the same vein, it is rather expected that intervention modulating motor learning and motor memory consolidation would involve neural circuits including M1. It does not necessarily imply that the neural substrates supporting the effect of these different interventions are similar.

Authors: The notion that M1 would be involved in motor learning (but maybe this is not so clear or obvious for consolidation) is a good point. Again, the critical point we are trying to make is that heightened M1 activity (especially at the time of test) seems to predict gain - and - this has been reported following sleep, exercise, and IP (indeed Tunovic et al. go so far as to argue that this is a neural signature for the onset of procedural consolidation). How this occurs (i.e., local effect directly at M1 or from remote activation) and, as noted by the reviewer, if this occurs in a similar manner across these interventions is indeed unclear. We have noted this important point explicitly in the manuscript and given credit to this reviewer in a footnote.

"It’s important to note these data do not necessarily imply that the neural substrates supporting the effects of these different interventions are (a) restricted to just M1, or (b) involve the same neural substrates. Indeed, there are recent data indicating that exogenous stimulation using tDCS of neural sites beyond M1 during RP can mediate the nature of offline changes in performance [14]. At this juncture then, the extant data suggest that, much like the case for sleep and exercise, the implementation of consolidation following IP that moves skill memory from its initial labile to more stable or even enhanced form is linked to heightened activity at M1 or maybe more broadly in neural circuitry incorporating M1. Deciphering if this occurs from local effects at M1 or from remote influences impinging on M1 and if these neural dynamics coincide across sleep, exercise, and practice organization will require additional experimental attention in the future.” This statement is used to conclude the section just prior to - Final Thoughts section.

Last, none of the limitations of CI experiments are mentioned in the opinion. This information will be of great interest for the reader. I would recommend adding a limitation section that highlights eg, how some of the previous CI research might have been confounded by eg. practice order effect (Sherwood & Lee, 2003).

Authors: Given this is an opinion piece we tried to be sensitive to the associated length limits. We do however think the reviewer is correct in noting that the impact of CI on learning is not universally demonstrated. We tried to make note of this by including what we think are two pertinent references addressing some of the limitations of this practice scheduling phenomenon. If there are more appropriate references we can of course include them.

Russell, D.M., & Newell, K.M. (2007) How Persistent and General Is the Contextual Interference Effect? Research Quarterly for Exercise and Sport, 78, 318-327.

Wulf, G., & Shea, C.H. (2002). Principles derived from the study of simple skills do not generalize to complex skill learning. Psychonomic Bulletin & Review, 9, 185-211.

Minor points:

There are multiple typos in the text. A few examples are listed below:

- Line 12: resulting is significant gains

Authors: Requested correction made

- Line 16: post-practice consolidation manifest as offline performance gain

Authors: Requested change has been incorporated into a re-working of the significance statement

- Line 211: attention to the nature of practice itself, in particular the manner in which practice is organized, may not be such a bad thing, as it to, can foster ...

Authors: This sentence has been rewritten and hopefully is a little clearer.

Some sentences are rather unclear and would benefit from revisions:

- Line 21: Offline gain from improved consolidation from offline influences such as sleep, exercise, as well as online manipulations such as interleaved rather than repetitive practice is related to increased cortical excitability in neural circuits that include M1.

Authors: As requested the significance statement was reworked and hopefully offers more clarity.

- Line 144: Mechanistically, consolidation that is assumed to be responsible for offline gain in novel motor skill, has been argued to be dependent on, or at least involve, neural circuitry that includes, but probably not limited to [e.g., see (Mirdamadi & Block, 2020)], the primary motor cortex (M1) (Robertson & Takacs, 2017)

Authors: This sentence has been re-written to improve clarity a requested by Reviewer 1.

REVIEWER 2:

This is a well-written, interesting opinion piece on potential similarities between conditions which promote offline learning... I just had a few question/observations.

Authors: Thanks for the positive comments regarding the manuscript.

• I think it would help to have clearer distinctions/clarity between practice interventions and post-practice interventions. It appears from the outset that the opinion paper is about post-practice interventions and that these are designed to augment the “learning” that took place during the practice episode and presumably is still continuing. An intervention such as sleep, rest, exercise or stimulation is therefore a supplement to the quality and/or quantity of the initial practice episode. When the idea of effective practice organization is introduced after discussion of post-practice interventions, this distinction then gets blurred (l63>).

Authors: Good point. We make an explicit note of the distinction between manipulating sleep/exercise as a direct manipulation of what occurs during the post-practice period vs. the delayed effect of what happens during this period by manipulating practice structure.

Specifically, we now state “In the present case, the potency of one particular practice organization for encouraging post-practice consolidation is highlighted by briefly reviewing some recent data addressing contextual interference (CI), a practice scheduling phenomenon that has a rich history in the motor skill literature [8]. Thus, rather than manipulate the post-practice period directly (i.e., by experiencing sleep or exercise after practice), here we demonstrate that that the arrangement of practice can be such that post-practice memory processing is encouraged that can lead to sizeable latent gains.”

• Related to this point of clarity above, is offline enhancement from “good quality” practice observed with other conditions of practice beyond CI? What is the critical ingredient that likely leads to offline gains? I appreciate the later discussion of M1 activation as mechanisms, but can the authors speculate on the actual method or quality of practice itself that is needed (perhaps beyond the CI effect)?

Authors: We originally tried to incorporate some other practice manipulations that might be noteworthy with respect to fostering consolidation. For example, there some nice recent work addressing mental imagery. However, this became a little distracting from the main message which is to highlight practice per se should be overlooked as a means of inducing this important memory process rather than to provide greater detail as to all of the practice manipulations that might be useful in accomplishing this outcome. In our opinion, the CI literature is the most developed to make this point.

• It might help the reader to give some description as to how offline gains and losses are calculated in a CI paradigm in view of the different types of practice and the fact that the end of the practice session reflect different sequences for IP and RP groups. These gains are still interesting, but is it because performance has not plateaued for the IP groups in comparison to the RP groups? Are offline gains still seen if the same level of accuracy is attained at the end of practice by these different groups? Are gains ever inferred from differences in immediate and delayed retention tests where the different groups are tested under the same conditions?

Authors: Ok, we took a shot at answering each of these issues in the text but it took quite a bit of space to develop. So we decided to not include the technical information regarding measurement. I should point out that in a number of these studies performance is similar at the end of practice (not always). In almost all of our work we use a common test at all test intervals (usually RP, so we load the design in favor of the RP condition. Yet IP still shows advantages.

We allude to this feature of our work when we state “Finally, it is critical to highlight that offline enhancement garnered via IP is not restricted to retention that is evaluated after 24-hrs or longer during which the learner is privy to overnight sleep as is the case for the influence of acute exercise for offline gain [4]. Performance facilitation from IP has also been observed during tests administered as early as 6-hr following the termination of practice as well as during the more common time frames used to assess memory, typically up to 72-hrs, when sleep-related consolidation might also contribute to any reported benefits [14]. These data are important because they implicate practice organization, specifically IP, as a means to mediate post-practice consolidation independent of the powerful impact induced from exposure to sleep.” This appears toward the end of the section entitled - Increasing interference during practice can facilitate consolidation of novel skill memories.

• In discussing the mechanistic similarities between post-practice unrelated task methods and practice methods themselves, is it simply the case that any method which excites M1 “sufficiently” in practice or post-practice will produce offline learning? Does offline learning occur for cognitive tasks that do not involve action components and presumably no M1?

Authors: This questions is similar to the one raised by Reviewer 1 who noted that M1 would likely be involved in some way for motor learning or retention. Moreover, the likelihood is that other regions might also be involved and used differentially during sleep, exercise, and IP. I don’t think we would be too far off base assuming this would be the case when learning non-motor tasks.

We tried to address this by stating “It’s important to note these data do not not necessarily imply that the neural substrates supporting the effects of these different interventions are (a) restricted to just M1, or (b) involve the same neural substrates. Indeed, there are recent data indicating that exogenous stimulation using tDCS of neural sites beyond M1 during RP can mediate the nature of offline changes in performance [14]. At this juncture then, the extant data suggest that, much like the case for sleep and exercise, the implementation of consolidation following IP that moves skill memory from its initial labile to more stable or even enhanced form is linked to heightened activity at M1 or maybe more broadly in neural circuitry incorporating M1. Deciphering if this occurs from local effects at M1 or from remote influences impinging on M1 and if these neural dynamics coincide across sleep, exercise, and practice organization will require additional experimental attention in the future.”

MINOR

l12; “in"

Authors: Requested correction made

l29 “a new skill” or “new skills"

Authors: Requested change “a new skill” made

l59-63... this is a long and difficult sentence

Authors: We agree! It now reads “While the use of sleep and exercise as adjuncts to practice continue to capture a larger part of the research community’s attention, the importance of practice itself, arguably the most critical determinant of the development of skill memory, has taken a relative backseat.” Hopefully, eliminating a section of this sentence makes this easier to read.

l294: include “hours” in the figure heading after 24-72.

Authors: Requested change made

Figure 1: It would be useful to explain what “test” means in the relationship figure between offline gain and cortical excitability. Is this excitability after 24 or 72 hours of not practising, just as an individual is getting ready to execute the sequences in a retention phase, or during the retention phase?

Authors: The requested information has been included in the Figure caption

L114: change “than” to “that"

Authors: Requested change was made

L133: “taken as a whole...”

Authors: Requested change was made

L133>136: this is also quite a long difficult sentence that would benefit from parsing/rephrasing.

Authors: We reworked this sentence to hopefully improve comprehension. It now reads “Taken as a whole, it is difficult to ignore the observation that interleaving practice of multiple novel skills and experiencing a period of sleep or exercise are remarkably effective means of fostering memory consolidation that improve long-term skill retention.”

L161: given that this is a new paragraph here, it would help to specify what “these data” refer to in the first line.

Authors: This now reads “The aforementioned data..” to clarify the specific data we are referencing in this section.

L164: when you say “learning” here, can you be more specific, do you mean practice or post practice?

Authors: Learning here should more accurately read “practice.” The sentence now reads “This claim is further supported by evidence of delayed performance enhancement via the direct upregulation of M1 using non-invasive stimulation during practice of a novel skill”

L190-193: can this sentence be parsed/simplified too?

Authors: This sentence has been simplified and now reads “Identifying training methods to foster successful long-term retention while also inducing some capability for generalization is central to research focused on the development of skill memory.”

L212: maybe structures is not the best word here as it can imply brain areas... perhaps methods or schedules?

Authors: We agree. This now reads “Despite these advances, the present discussion reminds us that increasing our awareness of how practice is organized, may not be such a bad thing, as it too, can foster consolidation of skill memory not afforded by other practice schedules.”

## References

[B1] Bonstrup M, Iturrate I, Thompson R, Cruciani G, Censor N, Cohen LG (2019) A rapid form of offline consolidation in skill learning. Curr Biol 29:1346–1351. 30930043 10.1016/j.cub.2019.02.049 PMC6482074

[B2] Chen J, Roig M, Wright DL (2020) Exercise reduces competition between procedural and declarative memory systems. eNeuro 7:ENEURO.0070-20.2020. 10.1523/ENEURO.0070-20.2020 PMC7405072 32616624

[B3] Hesseg RM, Gal C, Karni A (2016) Not quite there: skill consolidation in training by doing and observing. Learn Mem 23:189–194. 10.1101/lm.041228.115 27084926 PMC4836636

[B4] Jo JS, Chen J, Riechman S, Roig M, Wright DL (2019) The protective effects of acute cardiovascular exercise on the interference of procedural memory. Psychol Res 83:1543–1555. 29637259 10.1007/s00426-018-1005-8

[B5] Kim T, Wright DL (2020) Transcranial direct current stimulation (tDCS) of SMA complex impacts the effectiveness of interleaved and repetitive practice schedules. Neuroscience 435:58–72. 10.1016/j.neuroscience.2020.03.043 32243907

[B6] Kim T, Rhee J, Wright DL (2016) Allowing time to consolidate knowledge gained through random practice facilitates later novel motor sequence acquisition. Acta Psychol (Amst) 163:153–166. 26686835 10.1016/j.actpsy.2015.11.012

[B7] Kim T, Chen J, Verwey WB, Wright DL (2018) Improving novel motor learning through prior high contextual interference training. Acta Psychol (Amst) 182:55–64. 29136517 10.1016/j.actpsy.2017.11.005

[B8] Kim T, Kim H, Wright DL (2021) Improving consolidation by applying anodal transcranial direct current stimulation at primary motor cortex during repetitive practice. Neurobiol Learn Mem 178:107365. 33348047 10.1016/j.nlm.2020.107365

[B9] King BR, Hoedlmoser K, Hirschauer F, Dolfen N, Albouy G (2017) Sleeping on the motor engram: the multifaceted nature of sleep-realted motor memory consolidation. Neurosci Biobehav Rev 80:1–22. 28465166 10.1016/j.neubiorev.2017.04.026

[B10] Li Y, Wright DL (2000) An assessment of the attention demands during random- and blocked-practice schedules. Q J Exp Psychol A 53:591–606. 10.1080/713755890 10881620

[B11] Lin CH, Winstein CJ, Fisher BE, Wu AD (2010) Neural correlates of the contextual interference effects in motor learning: a transcranial magnetic stimulation investigation. J Mot Behav 42:223–232. 20570818 10.1080/00222895.2010.492720

[B12] Lin CH, Knowlton BJ, Chiang MC, Iacoboni M, Udompholkul P, Wu AD (2011) Brain-behavior correlates of optimizing learning through interleaved practice. Neuroimage 56:1758–1772. 21376126 10.1016/j.neuroimage.2011.02.066

[B13] Mirdamadi JL, Block HJ (2020) Somatosensory changes associated with motor skill learning. J Neurophysiol 123:1052–1062. 10.1152/jn.00497.2019 31995429

[B14] Ostadan F, Centeno C, Daloze J-F, Frenn M, Lundbye-Jensen J, Roig M (2016) Changes in corticospinal excitability during consolidation predict acute exercise-induced off-line gains in procedural memory. Neurobiol Learn Mem 136:196–203. 27773595 10.1016/j.nlm.2016.10.009

[B15] Reis J, Schambra HM, Cohen LG, Buch ER, Fritsch B, Zarahn E, Celnik PA, Krakauer JW (2009) Noninvasive cortical stimulation enhances motor skill acquisition over multiple days through an effect on consolidation. Proc Natl Acad Sci USA 106:1590–1595. 10.1073/pnas.0805413106 19164589 PMC2635787

[B16] Robertson EM, Takacs A (2017) Exercising control over memory consolidation. Trends Cogn Sci 21:310–312. 10.1016/j.tics.2017.03.001 28363680

[B17] Robertson EM, Pascual-Leone A, Miall RC (2004) Current concepts in procedural consolidation. Nat Rev Neurosci 5:576–582. 15208699 10.1038/nrn1426

[B18] Russell DM, Newell KM (2007) How persistent and general is the contextual interference effect? Res Q Exerc Sport 78:318–327. 17941536 10.1080/02701367.2007.10599429

[B19] Shea JB, Morgan RL (1979) Contextual interference effects on the acquisition, retention, and transfer of a motor skill. J Exp Psychol Hum Learn Mem 5:179–187.

[B20] Tunovic S, Press DZ, Robertson EM (2014) A physiological signal that prevents motor skill improvements during consolidation. J Neurosci 34:5302–5310. 24719108 10.1523/JNEUROSCI.3497-13.2014 PMC3983806

[B21] Wright DL, Verwey W, Buchanen J, Chen J, Rhee J, Immink M (2016) Consolidating behavioral and neurophysiologic findings to explain the influence of contextual interference during motor sequence learning. Psychon Bull Rev 23:1–21. 10.3758/s13423-015-0887-3 26084879

[B22] Wulf G, Shea CH (2002) Principles derived from the study of simple skills do not generalize to complex skill learning. Psychon Bull Rev 9:185–211. 10.3758/BF03196276 12120783

[B23] Xu W, De Carvalho F, Jackson A (2019) Sequential neural activity in primary motor cortex during sleep. J Neurosci 39:3698–3712. 10.1523/JNEUROSCI.1408-18.2019 30842250 PMC6510340

